# Supplementary material for: Jun Dimerization Protein 2 (JDP2) Increases p53 Transactivation by Decreasing MDM2
Source: Cancers (Basel). 2024 Feb 29;16(5):1000. doi: 10.3390/cancers16051000 (PMC10930919; doi:10.3390/cancers16051000)
Supplement: Supplementary file 1 [file cancers-16-01000-s001.zip › cancers-2883545-supplementary.pdf]

Figure 2A

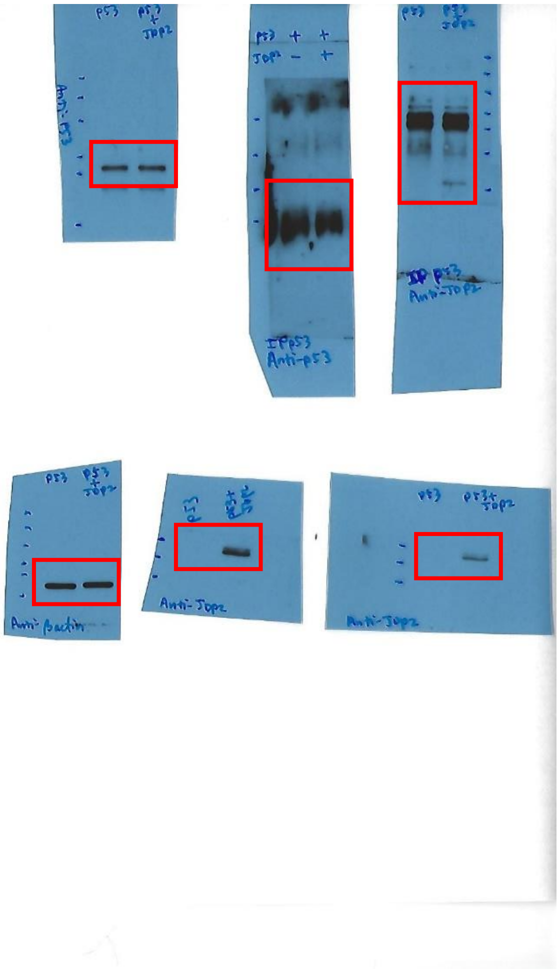

Figure 2B

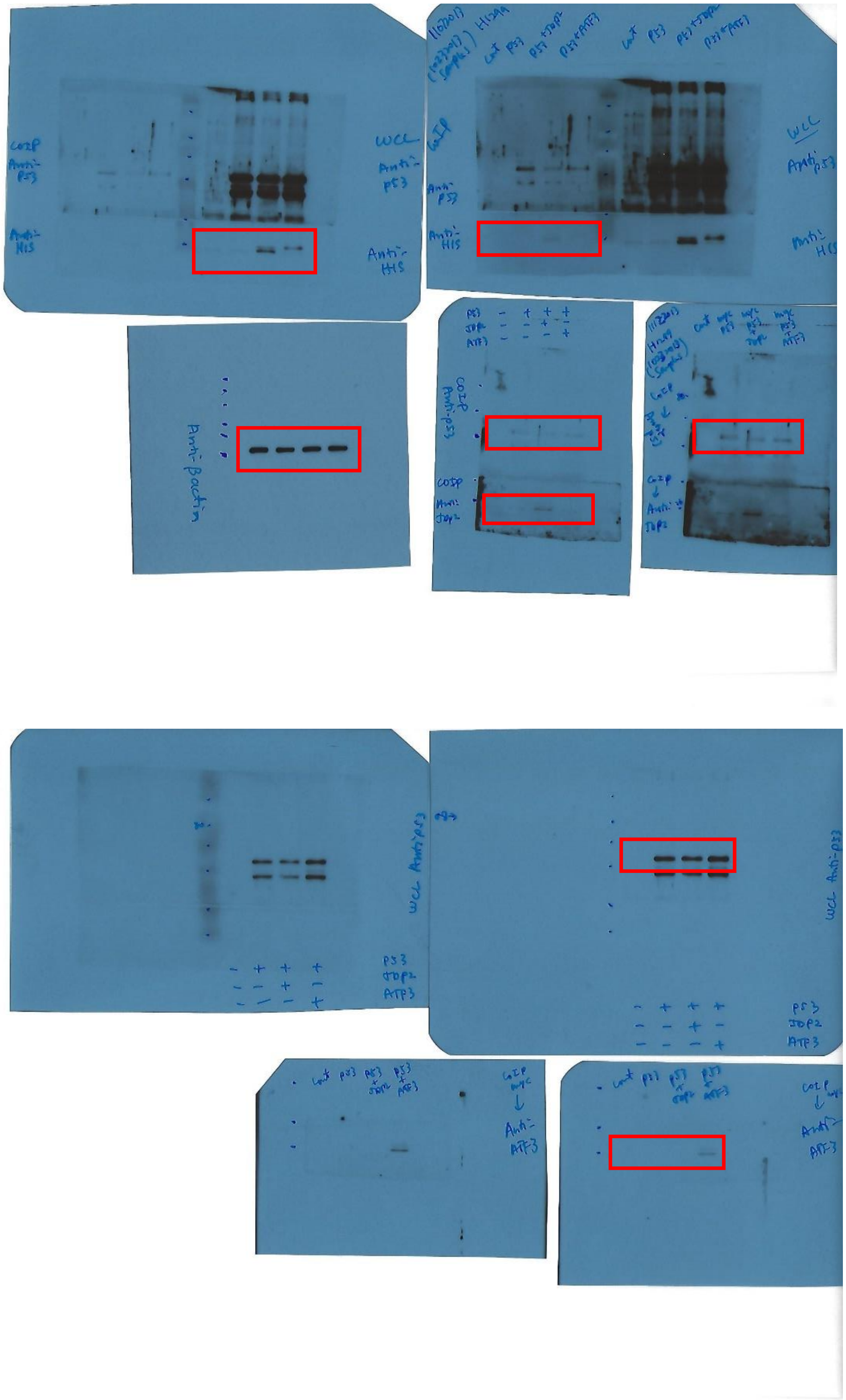
WCL
Anti-P53
Anti-HIS
Anti-β actin
P53
JDP2
ATF3
WCL Anti-P53
CoIP
Anti-ATF3

Figure 2C

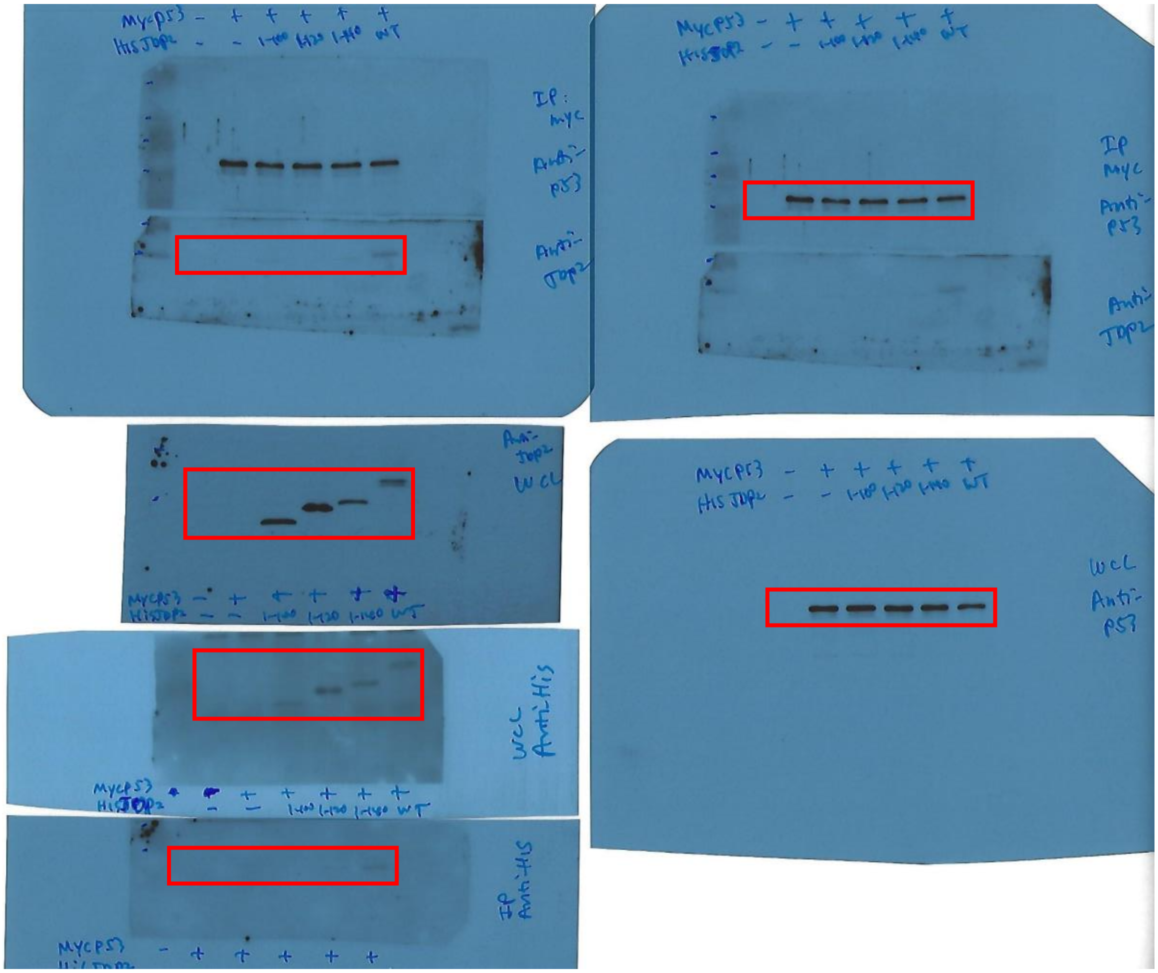
IP: myc
Anti-p53
Anti-TOP2
Anti-TOP2
WCL
WCL
Anti-His
IP
Anti-His
WCL
Anti-p53
WT

Figure 4A

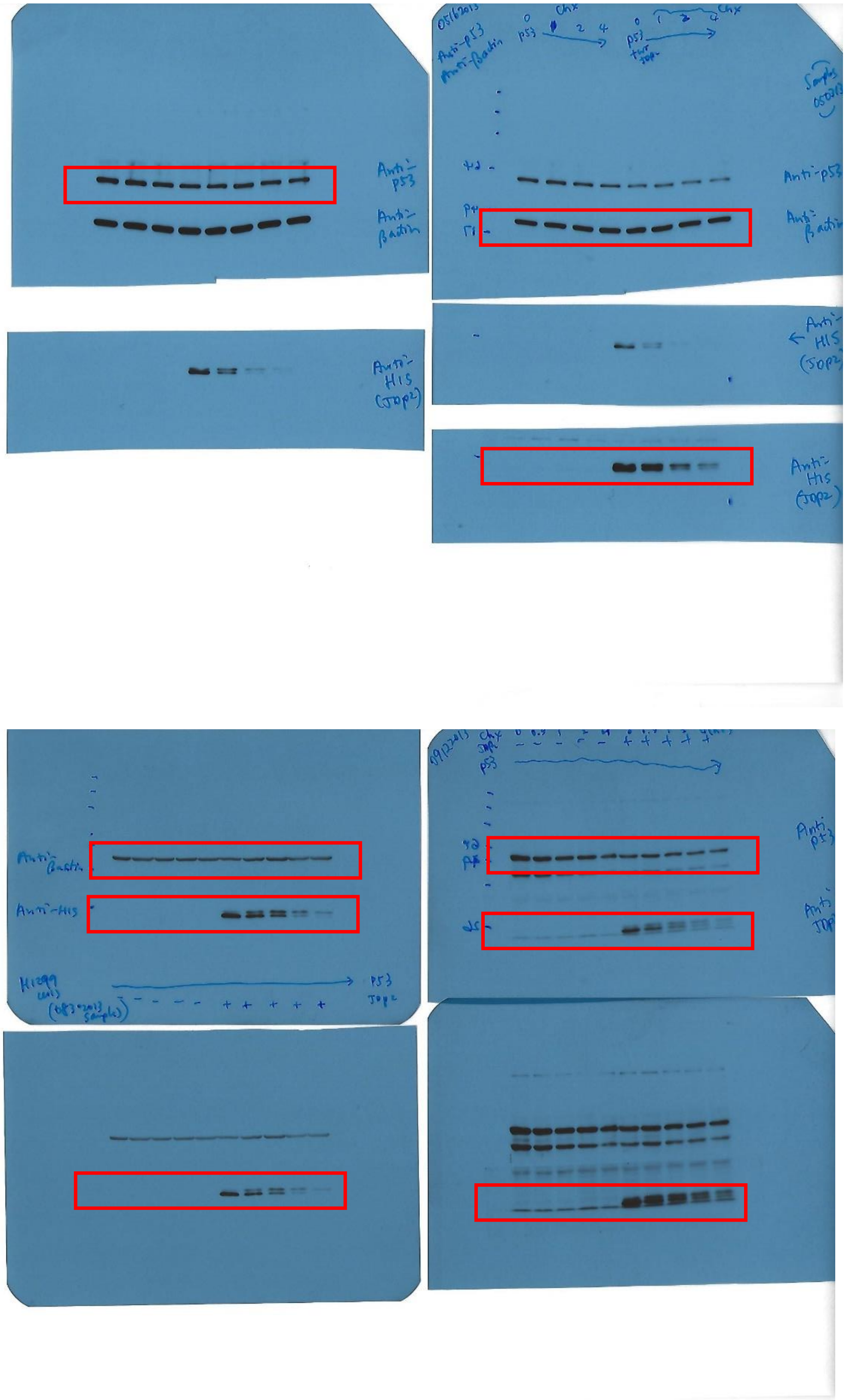
Anti-p53
Anti-βactin
Anti-HIS (JOP2)
Anti-p53
Anti-βactin
Anti-HIS (JOP2)
Anti-HIS (JOP2)
Anti-βactin
Anti-HIS
Anti-p53

Figure 5A

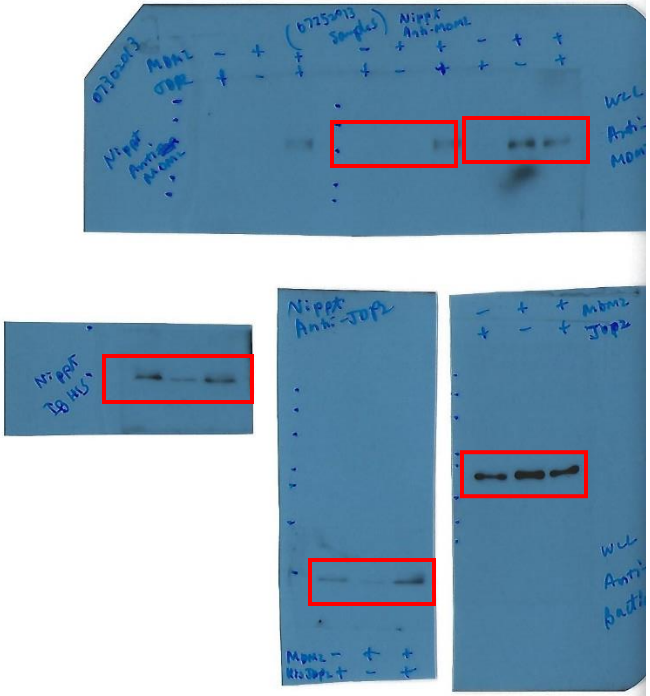

Figure 5B

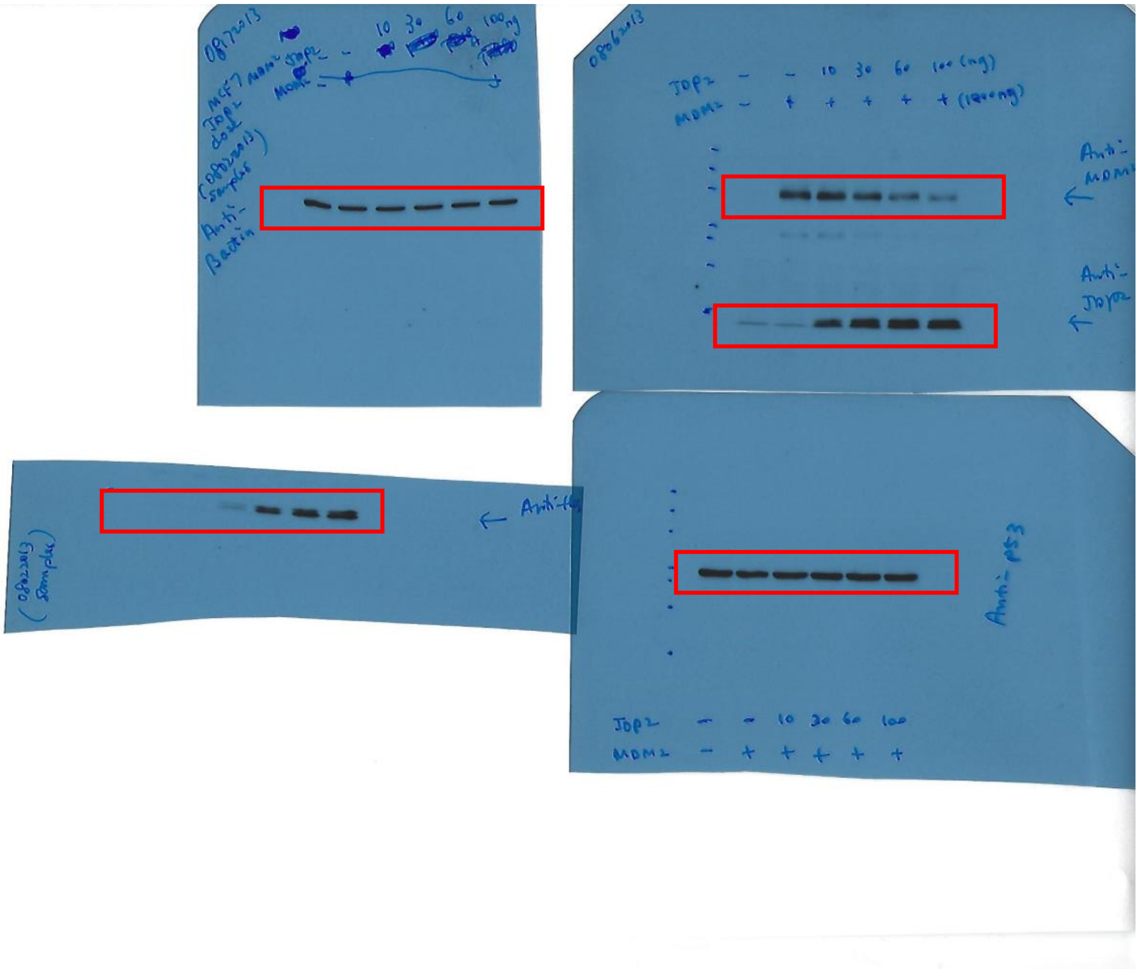

08062013
JOP2 – – 10 30 60 100 (ng)
MDM2 – + + + + + (1000ng)
Anti-MDM2
Anti-JOP2
Anti-Bactin
Anti-p53
JOP2 – – 10 30 60 100
MDM2 – + + + + +

Figure 5C

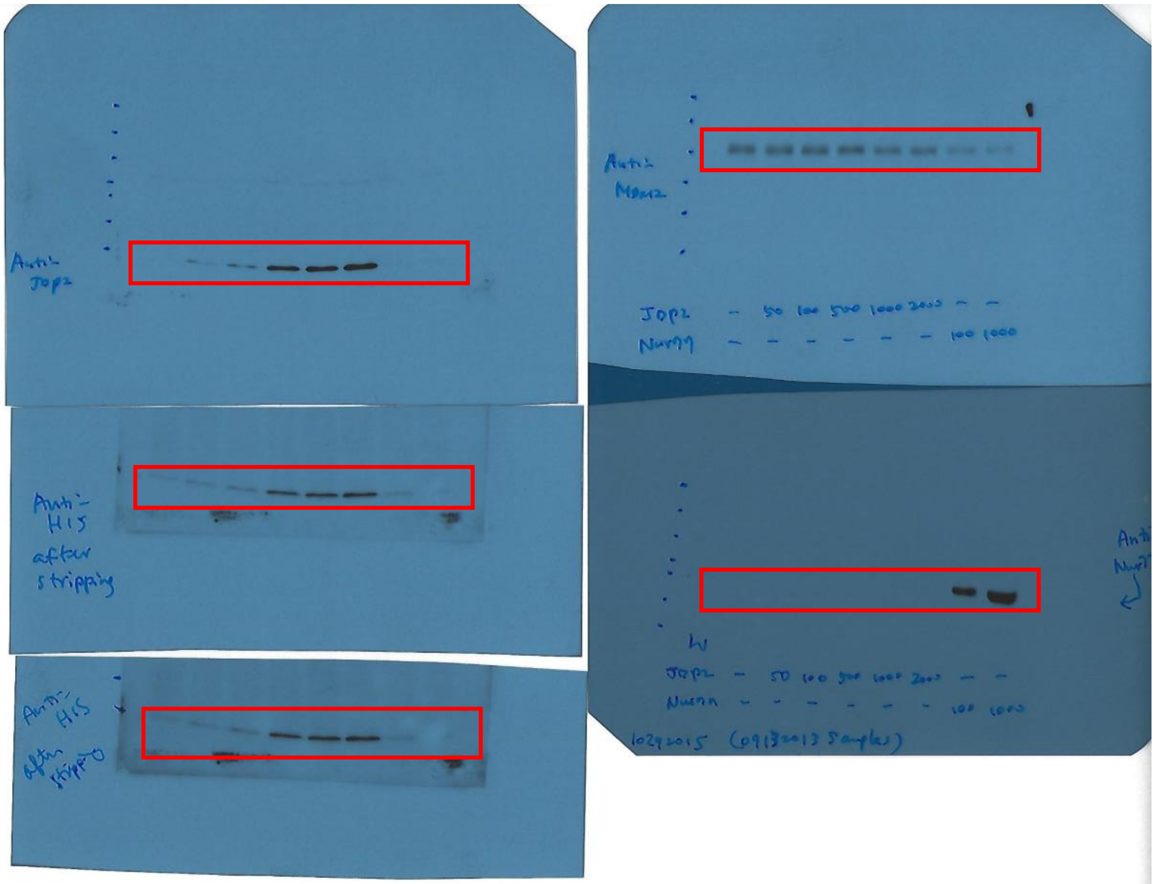
Anti-Jop2
Anti-HIS after stripping
Anti-HIS after stripping
Anti-Mdm2
Jop2 – 50 100 500 1000 2000 – –
Nur77 – – – – – – 100 1000
10292015 (09132015 Samples)

Figure 5D

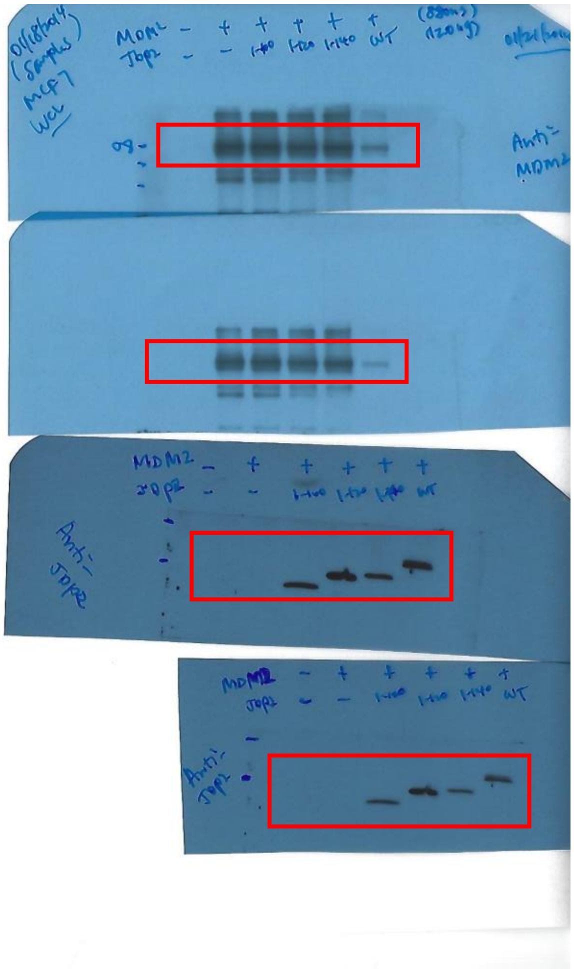

Figure 6

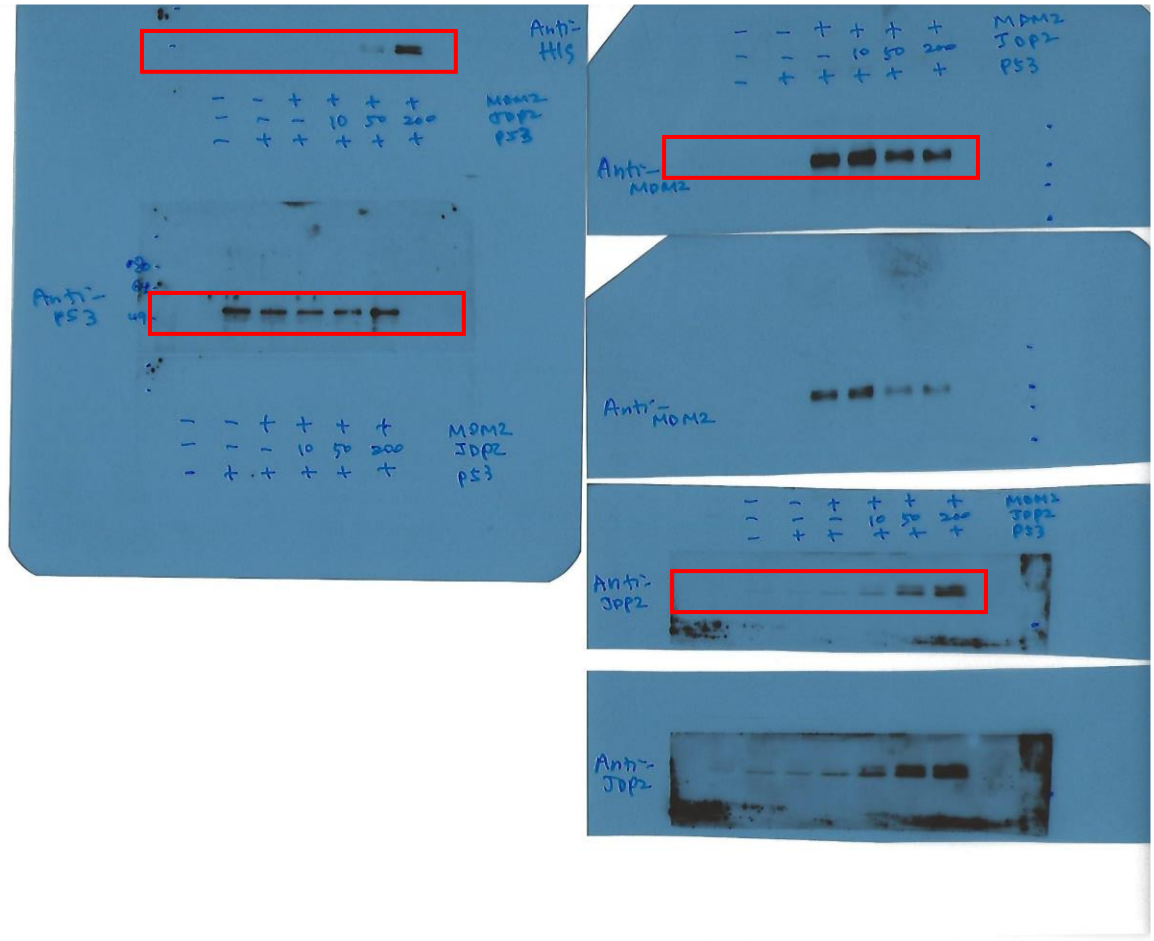

Figure 7B

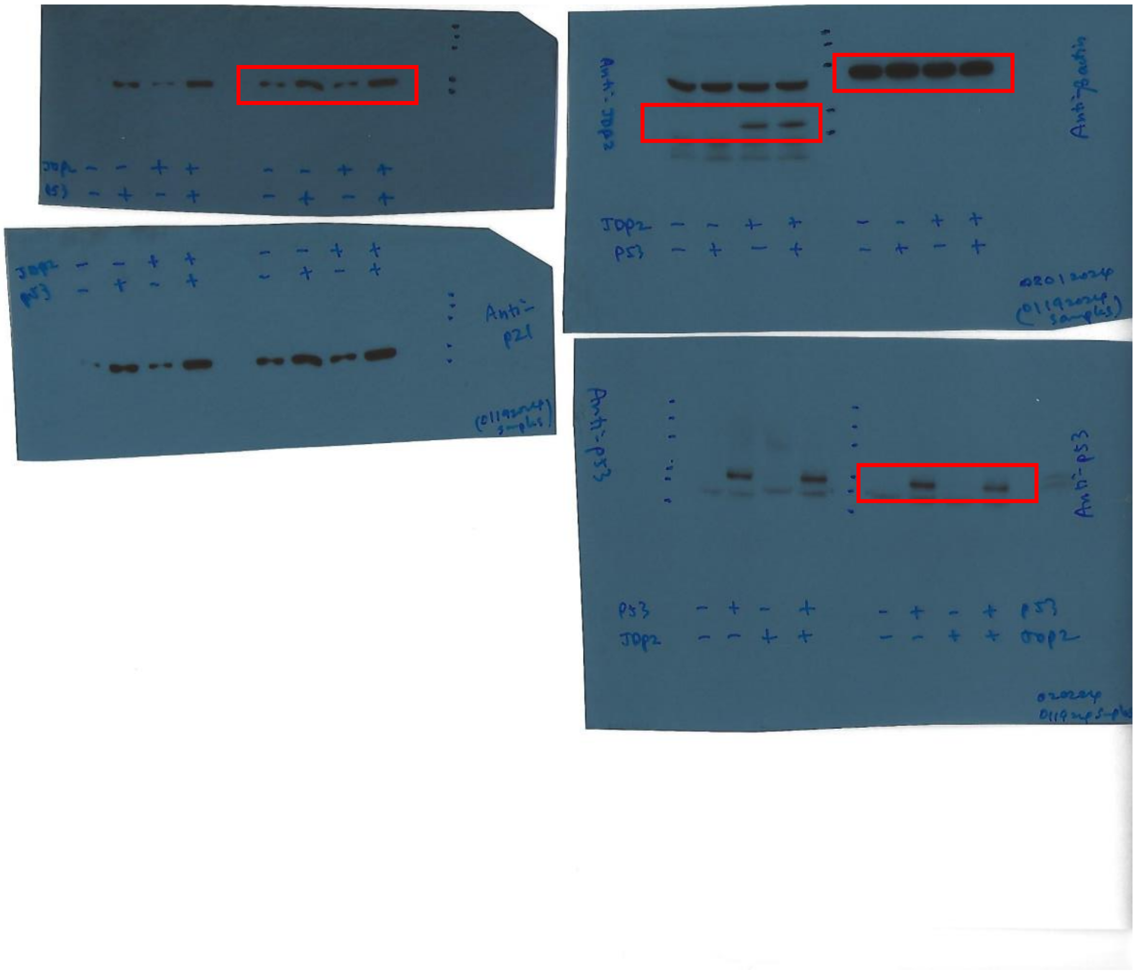

Figure S1. The original western blot figures.
